# Experimental pathogenesis of aquatic bird bornavirus 1 in Pekin ducks

**DOI:** 10.1038/s41598-023-45205-0

**Published:** 2023-10-23

**Authors:** Fernanda Ampuero, Alexander Leacy, Phuc H. Pham, Sunoh Che, Claire Jardine, Eva Nagy, Pauline Delnatte, Brandon N. Lillie, Leonardo Susta

**Affiliations:** Pathobiology Department, University of Guelph, 50 Stone Road East, Guelph, ON N1G 2W1 Canada

**Keywords:** Viral pathogenesis, Viral infection

## Abstract

Aquatic bird bornavirus 1 (ABBV-1) is a neurotropic virus that causes persistent infection in the nervous system of wild waterfowl. This study evaluated whether Pekin ducks, the most common waterfowl raised worldwide, are susceptible to ABBV-1 infection and associated disease. Groups of Pekin ducks were inoculated with ABBV-1 through the intracranial (IC; n, 32), intramuscular (IM; n, 30), and choanal (CH; n, 30) routes. Controls (CO; n, 29) received carrier only. At 1, 12, and 21 weeks postinfection (wpi), 7–14 birds were euthanized to assess virus distribution and lesions. Infection rates in the IC and IM groups were over 70%, while only 4 ducks in the CH group became infected. Neurological signs were observed in 8 ducks only, while over 25% of IC and IM birds had encephalitis and/or myelitis. Seroconversion was highest in the IC and IM groups, and mucosal ABBV-1 RNA shedding was most frequent in the IC group (53%). None of the fertile eggs laid during the experiment tested positive for ABBV-1 RNA. This study shows that Pekin ducks are permissive to ABBV-1 infection and partly susceptible to associated disease. While mucosal shedding may be an important route of transmission, congenital infection appears unlikely.

## Introduction

Aquatic bird bornavirus 1 (ABBV-1) belongs to the *Bornaviridae* family, *Orthobornavirus* genus, and *Orthobornavirus avisaquaticae* species^1^. It is a neurotropic virus that circulates in wild waterfowl and is closely related to the congeneric parrot bornaviruses (PaBVs), the causative agents of proventricular dilatation disease (PDD) in psittacine birds^2–5^. ABBV-1 was originally detected in Canada geese (*Branta canadensis*) and trumpeter swans (*Cygnus buccinator*) from Canada and the United States^6,7^. In these species, ABBV-1 has been associated with disease, as illustrated by a retrospective series of postmortem cases from the University of Guelph, which documented that infected birds had a history of neurological signs, and presented with poor body condition, upper gastrointestinal impaction, and variably severe mononuclear inflammation in the central, peripheral, and autonomic nervous systems^8^. However, since this was not a randomized controlled trial, a definitive causal relationship between infection and disease could not be made. Moreover, while ABBV-1 appears to be widespread in several species of the *Anseriformes* and *Charadriiformes* orders^9,10^, it has been also detected in distant taxa, such as a bald eagle (*Haliaeetus leucocephalus*)^11^ and an emu (*Dromaius novaehollandiae*) from a zoological collection^12^ suggesting the potential for a broad host range.

Recently, our research group isolated ABBV-1 from the brain of a naturally infected Canada goose^13^ and used it to inoculate chickens and Muscovy ducks (*Cairina moschata*)^14,15^. In both studies, birds became infected through the intramuscular and intracranial (but not oral) routes, although Muscovy ducks had a wider virus tissue distribution compared to chickens^14,15^. Additionally, despite minimal to no clinical signs, both species developed histologic lesions similar to those seen in naturally infected birds (i.e., mononuclear encephalitis and myelitis), confirming an association between ABBV-1 infection and inflammation of the nervous tissue^14,15^. Lack of neurological signs in experimentally infected birds is consistent with the asymptomatic infections often observed in natural ABBV-1 cases^7^, corroborating the notion that healthy waterfowl may be carriers of the virus.

In an effort to evaluate ABBV-1 host restriction and pathogenesis in waterfowl species, the goal of the present study was to assess the susceptibility of Pekin ducks (*Anas platyrhynchos* ssp. *domesticus*) to become infected with ABBV-1 and develop lesions and clinical signs, as well as their ability to shed the virus in the environment, taken as a proxy for transmission potential. Pekin ducks were chosen as an additional waterfowl representative distinct from Muscovy ducks (already evaluated in a previous study^14^), in order to describe susceptibility of infection among different domesticated anatid species with distinct geographical origin: the Americas (Muscovy duck) and Eurasia (Pekin duck)^16^. Moreover, Pekin ducks are closely related to mallards (*Anas platyrhynchos*), a common wild waterfowl from which Pekin ducks have been selected^16^. Lastly, the Pekin duck was used as it is the most common waterfowl species raised worldwide^17^, making the question of ABBV-1 susceptibility relevant under a production point of view.

## Results

### Clinical signs and gross findings

Pekin ducklings were inoculated with ABBV-1 through the intracranial (IC; n, 32; 10^4.16^ focus forming units [FFUs]/bird), intramuscular (IM; n, 30; 10^4.46^ FFUs / bird), and choanal (CH; n, 30; 10^4.46^ FFUs/bird) route, with the control (CO; n, 29) group receiving carrier only through all routes (Supplementary Fig. 1). Within 24 h after inoculation, 1/29 and 2/32 ducks from the CO and IC groups, respectively, were euthanized due to acute cerebral hemorrhage and excluded from the study.

Of the 90 ducks inoculated with ABBV-1, 1 IC (#272), 6 IM (#352, 353, 356, 377, 354, 371), and 1 CH (#476) bird showed neurological signs (head bobbing, ataxia, inability to stand), which occurred most frequently (7/8) between 52 and 78 days postinfection (dpi), while only one IM bird (#371) was affected at 124 dpi (1/8). All these birds tested positive for ABBV-1 RNA in the brain and/or spinal cord, with 7/8 (i.e., all except #371) presenting also microscopic lesions in the central nervous system (CNS). See below for description of relationship between clinical signs and infection parameters.

An additional 3 birds died or had severe clinical signs that warranted humane euthanasia; the cause of disease in these birds was identified as heterophilic bacterial meningitis (CH duck, 15 dpi), severe pulmonary aspergillosis (CH duck, 68 dpi), and septic arthritis (IC duck, 68 dpi). Of these, the CH duck with meningitis tested negative for ABBV-1 RNA in the CNS, while the other two were positive (see below). None of these birds had histologic lesions consistent with ABBV-1 infection, and their demise was considered incidental.

No duck in the entire experimental cohort had gross lesions attributable to ABBV-1 infection, such as proventricular dilatation or poor body condition, as assessed during necropsy.

### Histopathology

In total, 59 ducks underwent histological assessment in multiple tissues (as part of full sampling), and the brain and spinal cord from an additional 31 ducks were processed as part of partial sampling (Supplementary Fig. 1). Lesions suggestive of virus infection were mononuclear inflammation of the central and peripheral nervous tissue, and chromatolysis of neurons in the brainstem (Table 1).

**Table 1:** Mononuclear perivascular inflammation and neuronal chromatolysis tallied by weeks postinfection (wpi), as detected in different areas of the nervous tissue of Pekin ducks inoculated with aquatic bird bornavirus-1 (ABBV-1) through the intracranial (IC), intramuscular (IM), and choanal (CH) routes.

Site of inflammation	IC			IM			CH		
	1 wpi	12 wpi	21 wpi	1 wpi	12 wpi	21 wpi	1 wpi	12 wpi	21 wpi
Encephalitis	0/3*	7/9	1/14	0/3	6/8	5/14	0/3	0/9	1^^^/14
Cerebrum	0/3	7/9	1/14	0/3	6/8	4/14	0/3	0/9	1/14
Cerebellum	0/3	2/7	0/14	0/3	4/8	1/14	0/3	0/9	0/14
Brainstem	0/3	1/7	0/8	0/3	5/8	3/11	0/3	0/6	0/9
Myelitis	0/3	3/9	0/13	0/3	4/9	0/14	0/3	0/9	0/13
Peripheral neuritis	0/3	0/5	0/5	0/3	2/8	0/7	0/3	0/5	0/6
Chromatolysis**	0/3	1/7	0/8	0/3	2/8	0/11	0/3	1/6	0/9

*Indicates the number of tissues with lesions over the total tested for that time point.

**Chromatolysis was assessed in the brainstem only.

^Only one CH bird (#461) presented with inflammation.

#### Inflammatory lesions

**Figure 1:**
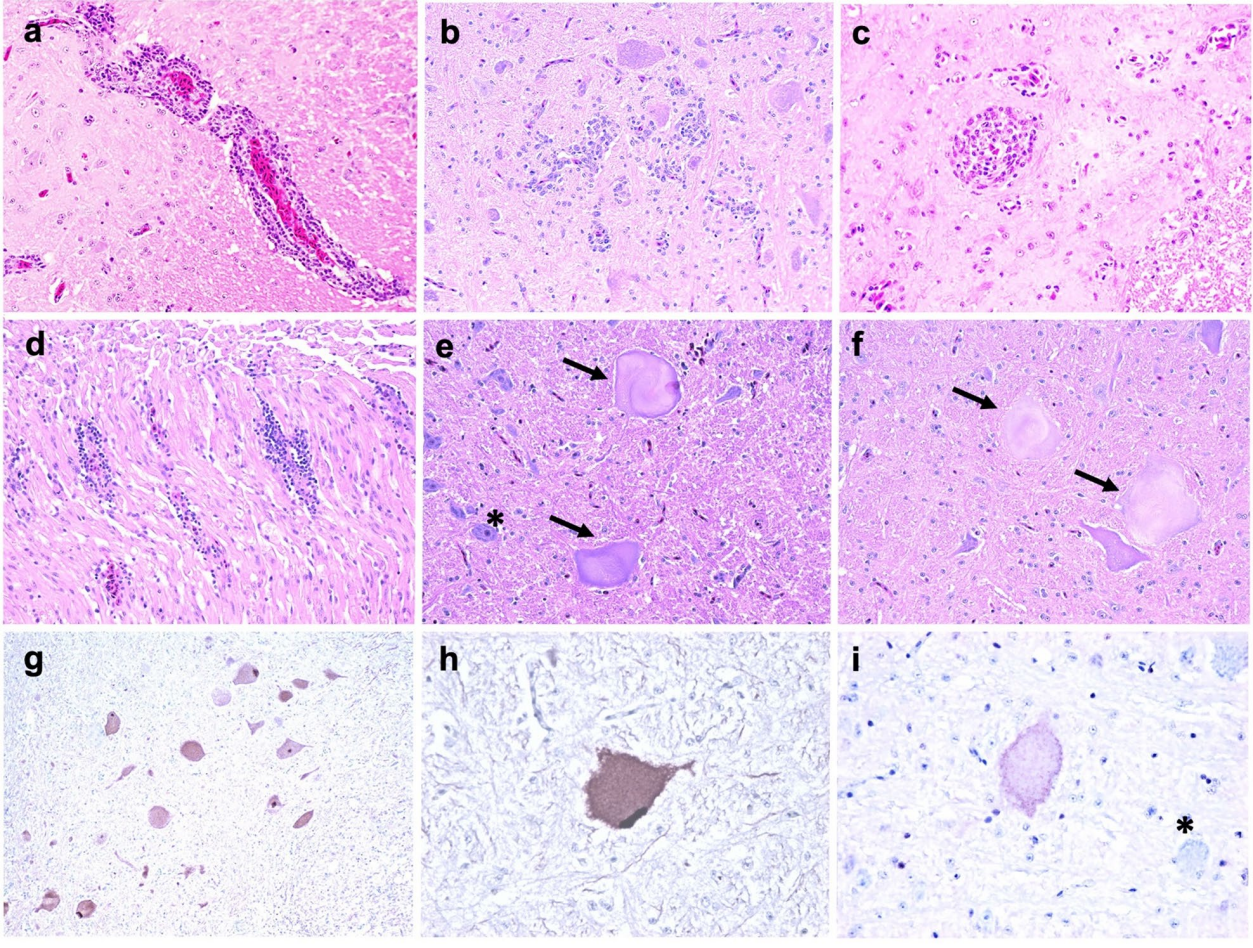
Microscopic lesions observed in Pekin ducks experimentally infected with aquatic bird bornavirus 1 (ABBV-1) through the intracranial (IC), intramuscular (IM), and choanal (CH) routes at 12 weeks postinfection. (**a**–**f**) Hematoxylin and eosin stain. (**g,h**) Immunohistochemistry for ABBV-1 N protein, DAB (3,3′-diaminobenzidine) chromogen with hematoxylin counterstain. (**a,b**) Perivascular cuffs in the brain of IC duck #271 (**a**) and IM duck #352 (**b**); original magnification × 20. (**c**) Perivascular cuffs in the grey matter of the spinal cord of IM duck #354. Original magnification × 40. (**d**) Lymphocytic perivascular cuffs in the sciatic nerve of IM duck #352. Original magnification × 20. (**e,f**) Brainstem of IC duck #272 (**e**) and CH duck #476 (**f**). Swollen neurons with marked chromatolysis (arrows) show loss of tigroid substance and inconspicuous nuclei; nonaffected neurons are shown by an asterisk (*). Original magnification × 40. (**g**) Brainstem of IC duck #272. Clusters of neurons in the brainstem are positive for ABBV-1 N protein, with both intracytoplasmic and nuclear reactivity; original magnification × 20. (**h,i**) Brainstem of IM ducks #356 (**h**) and #366 (**i**). When available on section, nuclei show strong reactivity (**h**), although cytoplasmic reactivity alone can be observed when nuclei are not captured on the plane of section (*indicates non affected neuron); original magnification × 40.

**Figure 2:**
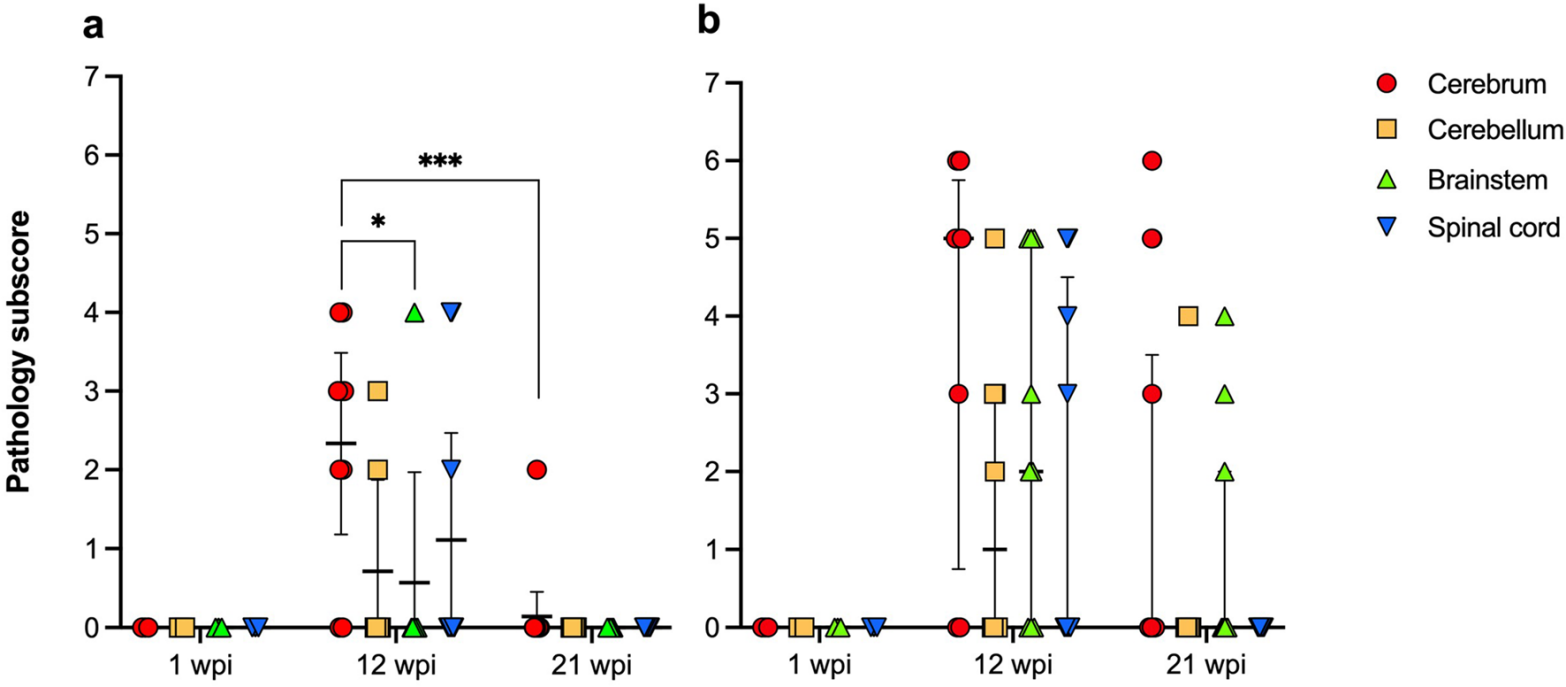
Pathology subscores for inflammation in different areas of the central nervous system of Pekin ducks experimentally inoculated with aquatic bird bornavirus-1 (ABBV-1) intracranially (**a**) and intramuscularly (**b**). Represented are data scatterplot with median and interquartile range. Significant differences are represented by binary connectors (Kruskal–Wallis test with Dunn’s correction for multiple comparisons; *< 0.05, **< 0.01, ***< 0.001, ****< 0.0001).

Perivascular cuffs were composed of mononuclear cells with scant cytoplasm and densely arranged chromatin (consistent with lymphocytes) and were randomly distributed throughout the gray and white matter of the cerebrum, cerebellum, brainstem, and spinal cord (Fig. 1a–c). In the CNS, mononuclear perivascular cuffs were present mainly in IC and IM ducks at 12 and 21 wpi, with only 1 CH duck (#461, the same bird with widespread infection and markedly positive serum) showing this type of lesion at 21 wpi (Table 1). All birds with inflammation were also positive for ABBV-1 RNA in the CNS. Frequency of inflammation peaked at 12 wpi, when most ducks in the IC (7/9; 77.8%) and IM (6/8; 75.0%) groups showed inflammation in the brain and spinal cord, and drastically declined by 21 wpi, when only 1 IC duck and 5 IM ducks had perivascular cuffs in the brain (Table 1). Similarly, lesions scoring showed that severity of inflammation in the CNS peaked at 12 wpi and decreased at 21 wpi (Fig. 2). In the IC group, the cerebrum at 12 wpi displayed a significantly higher score compared to the brainstem at the same time point, as well as the cerebrum at 21 wpi, when inflammation in other areas of the CNS was not observed (Fig. 2a). In the IM group, a decrease in inflammation severity was seen between 12 and 21 wpi, albeit non-significant, supporting a trend for decreasing inflammation over time (Fig. 2b).

Although inflammation was more common and severe in the cerebrum compared to other segments of the CNS in both the IC and IM groups at 12 and 21 wpi (Table 1 and Fig. 2), pairwise comparisons of proportions revealed no significant differences in the occurrence of inflammation between these CNS areas (p > 0.25).

Peripheral neuritis, characterized by lymphocytes around blood vessels and extending to the surrounding perineurium (Fig. 1d), was detected in the brachial plexus and / or sciatic nerve of only two IM ducks at 12 wpi (Table 1), a finding consistent with possible axonal virus trafficking.

#### Chromatolysis

One IC (#272), 2 IM (#356, 366), and 1 CH (#476) duck at 12 wpi showed multifocal neuronal chromatolysis in the brainstem (Tables 1 and 2). Affected neurons were characterized by markedly enlarged soma, up to 80 μm across, with loss of cytoplasmic basophilia and nucleus often pushed to the side (Fig. 1e,f). Immunohistochemistry on these 4 cases showed presence of ABBV-1 N protein in the nucleus (when available on section, due to the larger neuronal soma) and cytoplasm of the chromatolytic neurons in 3/4 birds (1 IC and 2 IM) (Fig. 1g–i). The CH duck showed no immunoreactivity in the CNS, consistent with the low burden of ABBV-1 RNA detected in the spinal cord of this bird (Table 2).

**Table 2:** Infection parameters in ducks inoculated with aquatic bird bornavirus 1 (ABBV-1) via the intracranial (IC), intramuscular (IM), and choanal (CH) routes, and which presented with neuronal chromatolysis or neurological signs.

Duck ID	Group	dpi^^^	Virus RNA copy number^^^^		Histologic lesions		IHC^§§^	ELISA^#^	Clinical signs
			Brain	Spinal cord	Inflammation^§^	Chromatolysis			
352	IM	52	5.72	6.59	Mild	−	+	−	Yes
353	IM	52	4.26	4.85	Severe	−	+	−	Yes
356	IM	61	3.92	7.29	–	+	+	−	Yes
377	IM	71	7.18	7.62	NA	NA	NA	+	Yes
354	IM	78	6.75	3.98	Moderate	−	+	NA	Yes
366	IM	84	6.93	7.08	Moderate	+	+	−	No
371	IM	124	0.61*	5.65	–	−	−	+	Yes
272	IC	61	7.33	7.29	Mild	+	+	−	Yes
476	CH	63	0.61*	1.91	–	+	−	(+)	Yes

*NA* indicates tissue/serum not available for testing.

^Indicates days postinfection (dpi).

^^Indicates the log(10) of RNA copy numbers per 150 ng of total RNA.

*Indicates samples negative by RT-qPCR (reported is limit of detection).

^§^Indicates inflammation as final score of the brain, divided as mild (score, 1–2), moderate (score, 3–4), and severe (score, 5–6).

^**§§**^Indicates immunohistochemistry (IHC).

^#^Indicates optical density values above the threshold (in parenthesis are values marginally above the threshold).

#### Incidental lesions

The three ducks that were euthanized due to aspergillosis (CH), septic arthritis (IC), and heterophilic meningitis (CH) did not present perivascular cuffs in either the CNS or PNS, and did not present neuronal chromatolysis.

### Virus distribution in tissues and environmental dispersal

#### Presence of ABBV-1 RNA in tissues

ABBV-1 RNA was quantified in the brain, spinal cord, proventriculus, kidney, gonads, and choanal and cloacal swabs of all ducks by reverse-transcriptase quantitative PCR (RT-qPCR). The frequency of positive tissues per experimental group and time point is reported in Table 3.

**Table 3:** Number of Pekin ducks inoculated with aquatic bird bornavirus-1 (ABBV-1) through the intracranial (IC), intramuscular (IM), and choanal (CH) routes that tested positive for ABBV-1 in select tissues at 1, 12, and 21 weeks postinfection (wpi).

Sample	IC			IM			CH		
	1 wpi	12 wpi	21 wpi	1 wpi	12 wpi	21 wpi	1 wpi	12 wpi	21 wpi
Brain	5/7*	9/9	14/14	0/7	8/9	13/14	0/7	1/9	2/14
Spinal cord	0/7	9/9	14/14	0/7	9/9	14/14	0/7	1/9	1^^^/14
Proventriculus	0/7	8/9	14/14	0/7	4/9	9/14	0/7	0/9	1/14
Kidney	0/7	8/9	14/14	0/7	4/9	7/14	0/7	0/9	1/14
Gonad	0/7	8/9	14/14	0/7	5/9	5/14	0/7	0/9	1/14
Oropharyngeal swab	0/7	5/9	11/14	0/7	0/9	3/14	0/7	0/9	1/13
Cloacal swab	0/7	0/9	1/14	0/7	0/9	0/14	0/7	0/9	0/11

*Indicates the number of positive tissues over the total tested for that time point.

^Only one CH bird (#461) had widespread ABBV-1 infection in multiple tissues.

**Figure 3:**
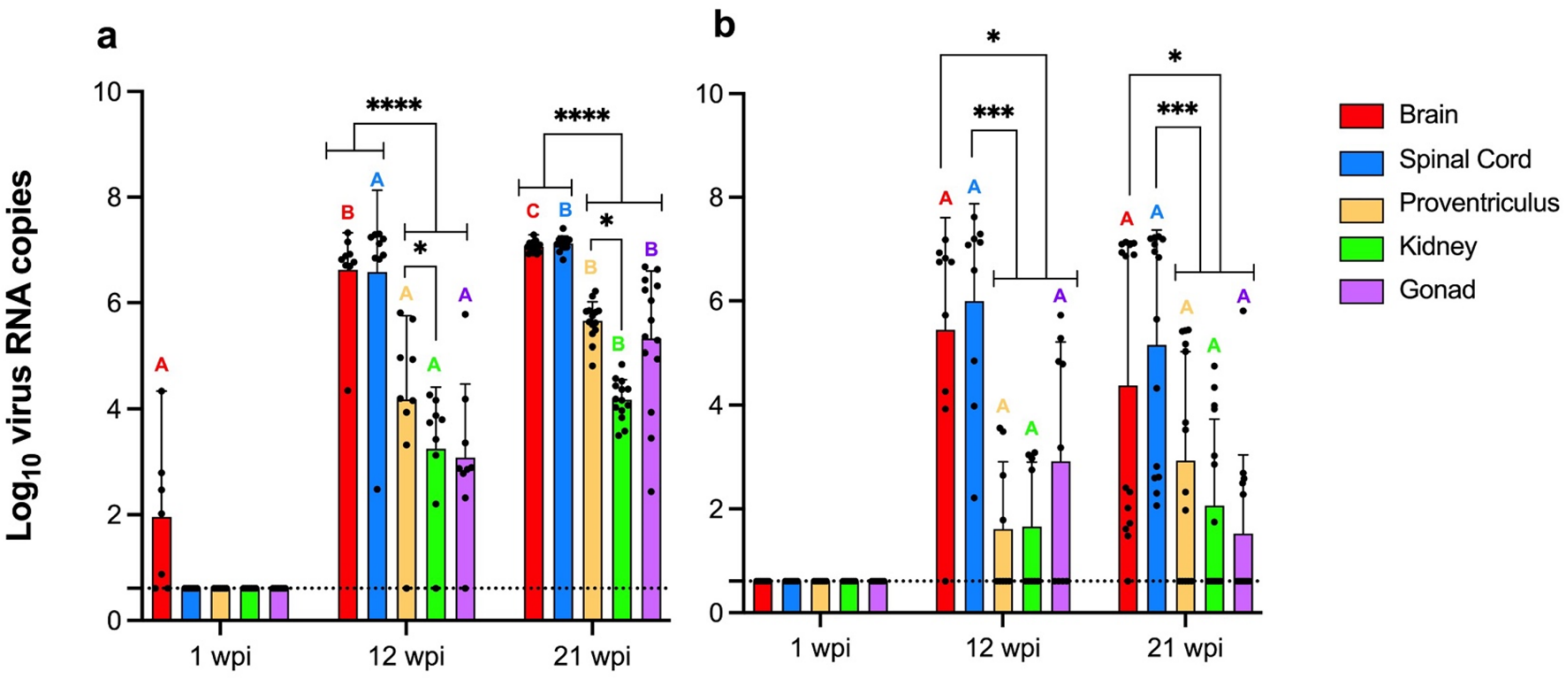
Magnitude of aquatic bird bornavirus-1 (ABBV-1) RNA copy number in tissues of Pekin ducks experimentally infected with ABBV-1 via the intracranial (**a**) and intramuscular (**b**) routes, and tested at 1, 12, and 21 weeks postinfection (wpi). Data columns represent mean log_10_ virus copies/150 µg total tissue RNA with standard deviation. Differences between group averages were tested by two-way ANOVA with Tukey’s test for multiple comparisons (*< 0.05, **< 0.01, ***< 0.001, ****< 0.0001). Significant differences between terms within the same time point are identified with binary connectors, and flat lines at the end of a connector indicate differences with multiple underlying terms (in that case, significance is reported for the higher value). Significant differences between the same tissue across time points are identified by color coded letters (p values not displayed). Negative samples (i.e., Ct value > 35) are represented at the limit of detection of the RT-qPCR assay (4.06 virus RNA copies per 150 ug of total tissue RNA; dotted line).

In the IC group, virus RNA was first detected at 1 week post-infection (wpi) in the brains of 5/7 (71.4%) birds, and by 12 and 21 wpi, 100.0% of brain and spinal cord samples tested positive. Visceral tissues (proventriculus, kidney, gonads) began testing positive starting at 12 wpi, and by 21 wpi all organs had ABBV-1 RNA (Table 3). Quantitative assessment showed an increase in the magnitude of virus RNA copies in all organs over time (Fig. 3a). These differences were significant for brain samples at 1 wpi compared to 12 and 21 wpi, brain samples between 12 and 21 wpi, and spinal cord samples between 12 and 21 wpi (p < 0.0001). A significant increase of ABBV-1 RNA concentration was also observed for proventriculus, kidney and gonad between 12 and 21 wpi (p < 0.0001). At every time point, brain and/or spinal cord had the highest virus RNA burden compared to the other tissues (p < 0.0001; Fig. 3a). Overall, a total of 28/30 (93.3%) birds became infected through the IC route, regardless of time point.

In the IM group, no tissues were positive for ABBV-1 RNA at 1 wpi, while by 12 wpi 100.0% of spinal cord and 88.9% of brain samples were positive, with similar high frequencies at 21 wpi. Visceral organs became positive by 12 wpi; however, the frequency of positive rates in these tissues was lower compared to the IC group, ranging between 44.4 to 55.5 and 35.7 to 64.3% at 12 and 21 wpi, respectively (Table 3). Similar to what observed for the IC group, virus RNA copies in the brain and spinal cord were highest compared to the other organs at 12 and 21 wpi (p < 0.05; Fig. 3b). While no significant differences were observed between the average virus RNA copies for the same organ between 12 and 21 wpi, a slight decrease was observed in the brain, spinal cord, and gonads at 21 compared to 12 wpi. This appeared to be associated with a higher data dispersal (Fig. 3b). Overall, a total of 23/30 (76.7%) birds became infected through the IM route, regardless of time point.

In the CH group, 4 birds tested positive for ABBV-1 RNA: 2 at 12 wpi (#455, brain; #476, spinal cord) and 2 at 21 wpi (#461, brain and spinal cord; #469, brain) (Table 3). At 12 wpi, 1 duck (#455) had low-level virus RNA copies in the brain only (10^1.3^ RNA copies/150 ng total RNA); this bird was euthanized because of pulmonary aspergillosis at 68 dpi. The other (#476) had low-level virus RNA copies in the spinal cord only (10^1.91^) and was euthanized due to progressively worsening ataxia at 63 dpi. The other 2 ducks were sampled at 21 wpi. One (#469) had low virus RNA burden in the brain only (10^2.32^), while the other (#461) was positive in the brain (10^6.89^), spinal cord (10^6.83^), and all visceral organs (proventriculus, 10^3.16^; kidney, 10^3.1^; gonad, 10^2.87^).

Blood was collected from 115/118 euthanized birds, and consistently tested negative for RNA of the ABBV-1 N gene by RT-qPCR (data not shown). No virus RNA was detected in any tissue of the CO group.

#### Presence of ABBV-1 nucleoprotein (N) in tissues

**Figure 4:**
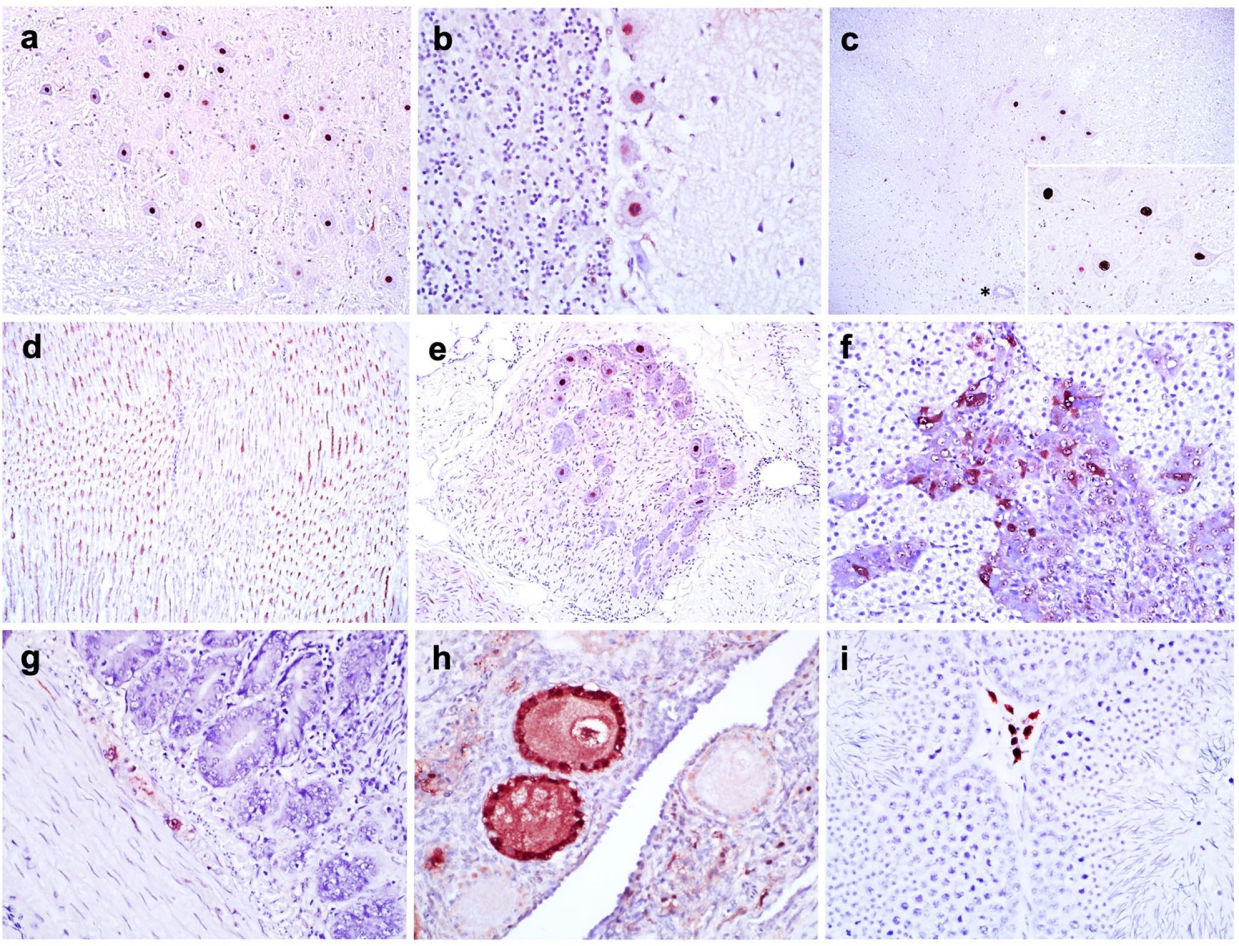
Immunohistochemistry for aquatic bird bornavirus 1 (ABBV-1) N protein was conducted to evaluate virus tissue distribution in Pekin ducks experimentally infected with ABBV-1 through the intracranial (IC), intramuscular (IM), and choanal (CH) routes, and sampled at 21 weeks postinfection. For all pictures, DAB (3,3′-diaminobenzidine) chromogen with hematoxylin counterstain. (**a**) Brainstem of IC duck #251. Presence of ABBV-1 antigen in the nuclei of neurons; original magnification × 20. (**b**) Cerebellum of CH duck #461. Presence of ABBV-1 antigen in the nuclei of Purkinje cells; original magnification × 40. (**c**) Spinal cord of IC duck #251. Presence of ABBV-1 antigen in the nuclei of neuronal bodies (inset). The central canal is indicated by an asterisk (*); original magnification × 10; inset, × 40. (**d**) Brachial plexus of IM duck #364. Presence of ABBV-1 antigen in axons; original magnification × 20. (**e**) Adrenal gland ganglia of IC duck #251. Presence of ABBV-1 antigen in the nuclei of neuronal bodies; original magnification × 20. (**f**) Adrenal gland of IC duck #251. Presence of ABBV-1 antigen in the nuclei and cytoplasm of scattered medullary cells; original magnification × 40. (**g**) Small intestine of IM duck #356. Presence of ABBV-1 antigen in the nuclei of neuronal bodies in the myenteric plexus; original magnification × 40. (**h**) Ovary of IC duck #282. Reactivity to ABBV-1 N protein is detected multifocally in the interstitial cells in the cortex, and within primordial follicles (including granulosa cells); original magnification × 40. (**i**) Testis of IC duck #269. Reactivity to ABBV-1 N protein is detected in scattered cells in the interstitium between seminiferous tubules); original magnification × 40.

Tested by immunohistochemistry (IHC) were 3 ducks that underwent full sampling from the IC, IM, and CH groups at 21 wpi, as well as the one CH duck (#461, partial sampling) that had tested positive in multiple tissues by RT-qPCR, and 1 CO duck from the same time point (negative control). Immunoreactivity was detected in 3/3 IC, 1/3 IM, and 1/4 CH birds. All birds positive by IHC in at least one tissue also showed presence of virus RNA in the CNS, except for one IM duck (#371), which was positive exclusively in the spinal cord by RT-qPCR but showed no immunoreactivity (Table 4). For the IC and IM birds, reactivity was present in 10/20 and 9/20 tissues. For 3 CH birds, no tissues were immunoreactive, while for the only CH bird with evidence of widespread infection by RT-qPCR, 5/8 tissues were immunoreactive. When positive, tissues had similar reactivity patterns across groups. In the CNS, immunoreactivity was seen in the nuclei of neurons randomly throughout the brain with no areas being more affected, Purkinje cells in the cerebellum, and in the grey matter of the spinal cord (Fig. 4a–c); while reactivity was not observed in glial cells, occasional ependymal cells in the choroid plexuses showed nuclear and/or cytoplasmic reactivity. In the peripheral nervous system (PNS), immunoreactivity was observed in nerve fibers of the brachial plexus and/or sciatic nerve (Fig. 4d), in the nuclei of neuronal bodies in ganglia adjacent to the adrenal gland (Fig. 4e) and lungs, as well as in the nucleus and cytoplasm of cells in the adrenal medulla (Fig. 4f). In the gastrointestinal tract, immunoreactivity was detected in the intramural plexuses of the proventriculus and various segments of the intestine (Fig. 4g), but not in the ventriculus. The CO duck showed no immunolabelling in any of the examined tissues (Table 4). Moreover, IHC was used to evaluate ABBV-1 N protein expression in the gonads of all the IC birds at 21 wpi, and in the CNS of animals with neurological signs. Results are separately reported the respective sections below.

**Table 4:** Tissue immunoreactivity for aquatic bird bornavirus 1 (ABBV-1) N protein in Pekin ducks inoculated with ABBV-1 via the intracranial (IC), intramuscular (IM), and choanal (CH) routes, as well as the control (CO), and sampled at 21 weeks postinfection.

Tissues	151 CO	251 IC	260 IC	268 IC	351 IM	364 IM	371 IM	453 CH	459 CH	464 CH	461* CH	Cells positive for ABBV-1 N antigen
Cerebrum	−	** + **	** + **	** + **	** + **	−	−	−	−	−	** + **	Neurons, Purkinje cells, ependymal cells
Cerebellum	−	** + **	** + **	** + **	** + **	−	−	−	−	−	** + **	
Brainstem	−	** + **	** + **	** + **	** + **	NA	−	−	−	−	** + **	
Spinal cord	−	** + **	** + **	** + **	** + **	−	−	−	−	−	** + **	Neurons
Peripheral nerves	−	−	−	** + **	** + **	** + **	−	−	−	−	NA	Nerve fibers of the brachial plexus
Kidney	−	−	−	−	−	−	−	−	−	−	−	
Gonad**	− (M)	+ (F)	+ (F)	+ (F)	− (M)	− (F)	− (F)	− (M)	− (F)	− (M)	+ (F)	Interstitial cells in the cortex, theca, granulosa, rare follicles
Proventriculus	−	** + **	** + **	** + **	** + **	−	−	−	−	−	−	Myenteric plexuses
Ventriculus	−	−	−	−	−	−	−	−	−	−	−	
Small intestine	−	** + **	** + **	** + **	** + **	−	−	−	−	−	NA	Myenteric plexuses
Large intestine	−	** + **	** + **	** + **	** + **	−	−	−	−	−	NA	
Pancreas	−	−	−	−	−	−	−	−	−	−	NA	
Liver	−	−	−	−	−	−	−	−	−	−	NA	
Heart	−	−	−	−	−	−	−	−	−	−	NA	
Thymus	−	−	−	−	−	−	−	−	−	−	NA	
Spleen	−	−	−	−	−	−	−	−	−	−	NA	
Bursa	−	−	−	−	−	−	−	−	−	−	NA	
Thyroid/parathyroid	−	−	−	−	−	−	−	−	−	−	NA	
Adrenal	−	** + **	** + **	** + **	** + **	−	−	−	−	−	NA	Medullary cells, neurons in adjacent ganglia
Trachea	−	−	−	−	−	−	−	−	−	−	NA	
Lung	−	−	−	−	−	−	−	−	−	−	NA	

NA indicates missing tissues. For bird #461, only 8 tissues were present, as the bird underwent partial sampling.

M or F indicates male or female.

*Indicates the bird number.

**See also Table 7.

#### Virus shedding and environmental dispersal

**Figure 5:**
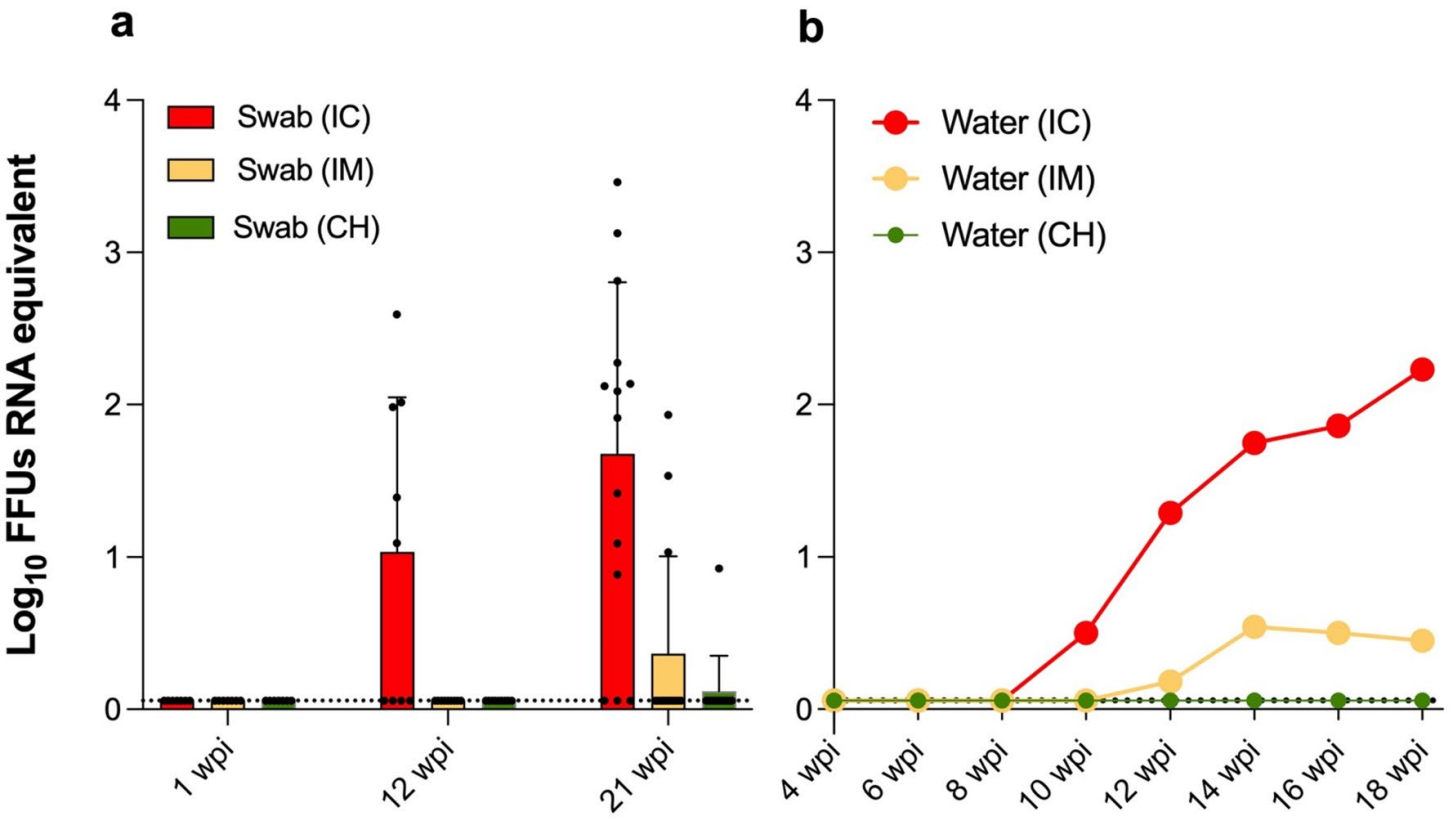
Log10 virus focus forming units (FFUs) RNA equivalent/84 μl of liquid, as assessed in oropharyngeal swabs (**a**) and drinking water (**b**) collected from Pekin ducks inoculated with aquatic bird bornavirus-1 (ABBV-1) intracranially (IC), intramuscularly (IM), or via the choanal slit (CH). Swabs were collected during necropsy at 1, 12, and 21 weeks postinfection (wpi). Water was sampled every 2 weeks from 4 to 18 wpi. Swab values are plotted as scatterplot with boxes representing mean with standard deviation. For water, single values are plotted over time. Negative samples are plotted at the limit of detection of the RT-qPCR assay (1.14 FFUs RNA equivalent/84 μl).

Virus dispersal was determined by quantification of virus RNA copies by RT-qPCR in the oropharyngeal and cloacal swabs at the time of euthanasia, as well as in the drinking water collected bi-weekly. Virus RNA was detected in the oropharyngeal swabs of 5/9 (55.5%) IC ducks at 12 wpi, and 11/14 (78.6%) and 3/14 (21.4%) IC and IM ducks, respectively, at 21 wpi. The magnitude of virus RNA in the oropharyngeal swabs was highest in the IC group at 21 wpi (Fig. 5a). Only one IC duck at 12 wpi had both oropharyngeal and cloacal swabs positive; all other cloacal swabs in the entire experiment tested negative. In the CH group, only 1 oropharyngeal swab tested positive at 21 wpi, although this bird (#455) was negative for ABBV-1 RNA in the other tested organs (Table 3). Except for the latter bird, all ducks that tested positive in the swabs were also positive for ABBV-1 RNA in the CNS and variably in the visceral tissues.

Viral RNA was detected in the drinking water starting at 8 wpi in the IC group, and 12 wpi in the IM group. The amount of virus RNA in the drinking water progressively increased over time in the IC group (Fig. 5b), consistent with the highest frequency of RNA shedding being at 21 wpi in the IC group. In the IM group, the amount of virus RNA in the drinking water peaked at 14 wpi, although it remained approximately 1–1.5 orders of magnitude less concentrated compared to the IC group. No viral RNA was detected in the drinking water of the CO and CH groups (Fig. 5b).

### Serology

**Figure 6:**
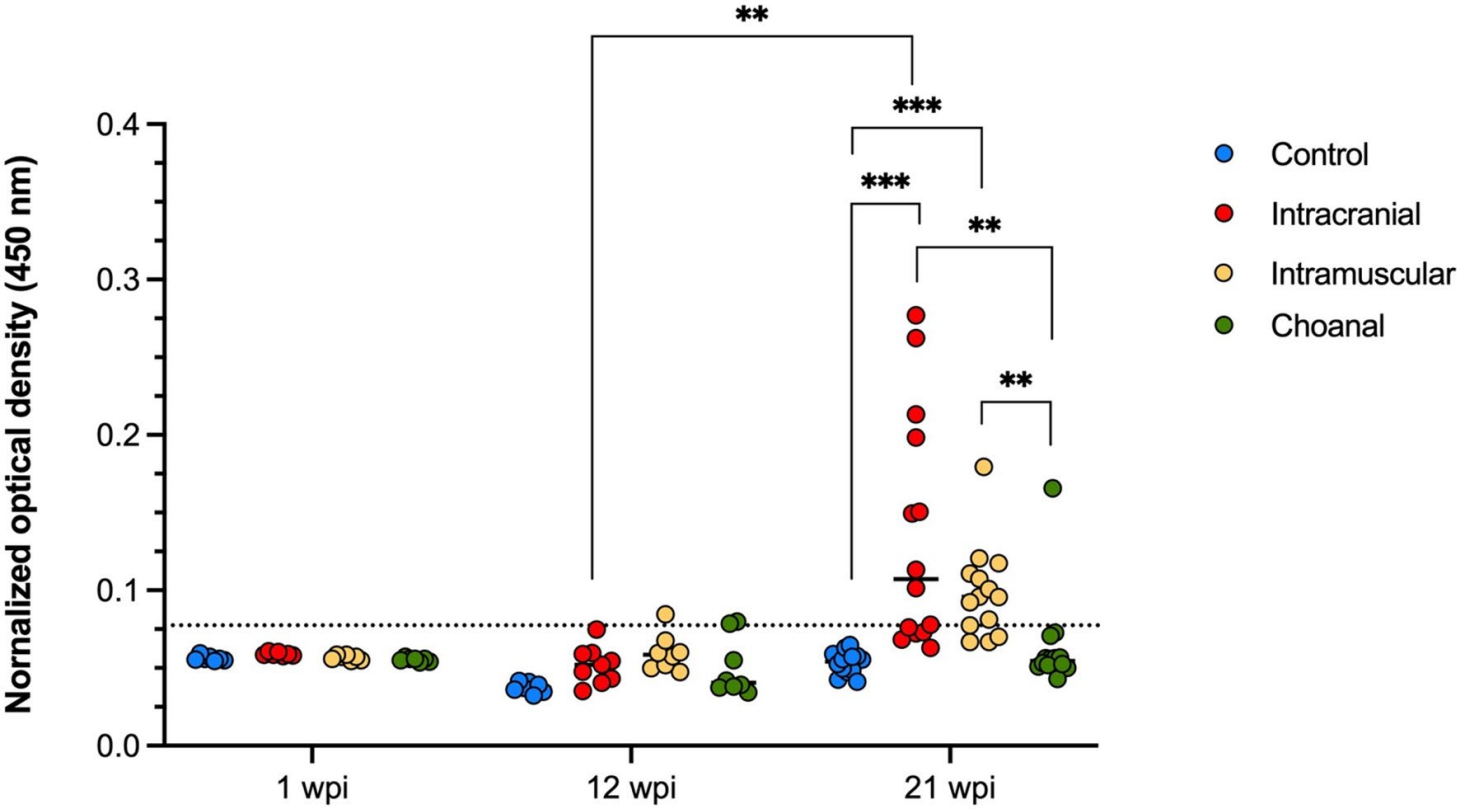
Normalized optical density (OD) derived from an ELISA assay against the ABBV-1 N protein and run using the sera of Pekin ducks inoculated with aquatic bird bornavirus-1 (ABBV-1) through the intracranial, intramuscular, and choanal routes. Sera were sampled at the time of necropsy at 1, 12, and 21 weeks postinfection (wpi). Data are represented as scatterplot. Differences between group averages were tested by Kruskal–Wallis test with Dunn’s test for multiple comparisons (*< 0.05, **< 0.01, ***< 0.001, **** < 0.0001). Significant differences between terms at the same time point, or between the same group across time points are identified by binary connectors. The threshold (OD = 0.077; dotted line) was calculated as the average between all negative control ducks (from all sampling points) plus three times the standard deviation.

All serum samples from ducks at 1 wpi had optical density (OD) values below the threshold. By 12 wpi, 1/8 (12.5%) IM and 2/8 (25.0%) CH ducks has serum samples with OD values marginally above the threshold. At 21 wpi, 9/14 (64.3%) IC, 10/14 (71.4%) IM, and 1/14 (7.1%) CH ducks had serum OD values clearly above the threshold (Fig. 6). The only positive CH duck at 21 wpi had a much higher optical density (OD) value compared to the other positive CH birds, consistent with being the only CH bird with widespread ABBV-1 RNA distribution in the CNS and visceral tissues (bird #461). The IC and IM birds at 21 wpi had the highest average OD values, which was significantly higher compared to both the CO and CH groups at the same time point, although there were no significant differences between the IC and the IM groups (Fig. 6).

### Relationship between clinical signs, infectious status, lesions, and serology

#### Descriptive statistics

The infection and pathology parameters of birds displaying neurological signs are reported in Table 2. All the 8 ducks with clinical signs were positive for ABBV-1 RNA in the brain and/or spinal cord, and IHC demonstrated presence of N protein in neurons in the brain and spinal cord of 5/8 (62.5%) birds. Inflammation was observed in 4/8 (50.0%) (mild, 2/4; moderate, 1/4; severe, 1/4), chromatolysis in 3/8 (37.5%), and positive sera in 3/8 (37.5%) (Table 2).

#### Multivariable logistic regression analysis

A multivariable logistic regression analysis was used to predict the occurrence of neurological signs, using as explanatory variables time postinfection, inflammation, virus RNA copies in the CNS, serology, and inoculation route. Based on the unconditional association results, cerebrum subscore, spinal cord subscore, and time postinfection were fed into a multivariable regression model, which showed that only time postinfection was significantly associated. Specifically, ducks at 12 wpi had 1.3-time higher odds (1.01–1.67 CI) of developing neurological signs compared to those at 21 wpi (p = 0.042).

Inflammation of the CNS was also predicted using a multivariable logistic regression analysis, using time postinfection, virus RNA copies in brain and spinal cord, serology, and inoculation route as explanatory variables. Based on the unconditional association results, virus RNA copies in the brain and time postinfection were fed into the multivariable regression model. Results indicated that both variables were significantly correlated with the outcome variable (Table 5). Ducks at 12 wpi displayed 1.25 times higher odds of having CNS inflammation than those at 21 wpi, while per each 10-time increase of RNA copies in the brain, the odds of CNS inflammation were 1.81 times higher.

**Table 5:** Multivariable logistic regression analysis used to predict the occurrence of inflammation in the central nervous system (dependent, categorical variable; presence/absence) in Pekin ducks experimentally inoculated with aquatic bird bornavirus 1 (ABBV-1) through the intracranial (IC), intramuscular (IM), and choanal (CH) routes.

Predictor	Value	Odds ratio	Std. err.	p value	95% Confidence interval
Virus copy numbers in the brain*	− 5 to 7.33	1.81	0.30	< 0.001	1.32–2.52
wpi	21 wpi	0.75	0.07	0.002	0.64–0.91
	12 wpi	Reference			

The explanatory (independent) variables used were week postinfection (wpi; categorical binary) and virus RNA concentration (RNA copies/150 ng total RNA; continuous).

*The virus RNA concentration was expressed as a log10 copy number per 150 ng of total RNA, and any zero values were changed to 1.0 × 10^–5^.

### Assessment of vertical transmission

#### Egg laying and infectious status of embryos

Eighty-eight, 60, 34, and 61 eggs were collected from the CO, IC, IM, and CH groups between 115 and 148 dpi, of which respectively 44.3, 45.0, 35.3, and 55.7% developed an embryo (Table 6, Supplementary Table 1). The ratio of fertile to total eggs laid, as well as the total number of eggs normalized to the number of hens were not statistically different between groups, as tested by Chi-square test (p = 0.26) and Kruskal–Wallis test with Dunn’s test for multiple comparisons (p = 0.195), respectively.

**Table 6:** Total number of eggs discarded and incubated, and samples obtained from incubated eggs, from each group of Pekin ducks inoculated with aquatic bird bornavirus 1 (ABBV-1) through the intracranial (IC), intramuscular (IM), and choanal (CH) routes, and the control (CO) group.

Destination and samples	CO	IC	IM	CH
Discarded	49	33	22	27
Incubated	39	27	12	34
Entire embryo	1	1	1	4
Brain only	2	0	0	1
Brain + pooled organs	35	26	11	29

A total of 111 embryos were tested, for which 104 brains, 101 pooled organs, and 3 entire early-stage embryos were available (Table 6); none of the tissues from embryos tested positive by RT-qPCR.

#### Infectious status of gonads

As egg parentage could not be determined for single eggs, we sought to evaluate the infection status of the gonadal tissue present at 21 wpi, since eggs were laid exclusively after 12 wpi. Overall, 100.0%, 20.0%, 16.7%, and 0% of ovaries, and 100.0%, 44.4%, 0%, and 0% of testis were positive for ABBV-1 RNA in the IC, IM, CH, and CO groups, respectively (Supplementary Table 2). Given the higher rate of gonadal infection in the IC group, we tested expression of ABBV-1 N protein by IHC in these tissues. When available for histological evaluation, all ovaries presented active folliculogenesis, and all testes active spermatogenesis. By IHC, ABBV-1 N protein was present in 8/8 (100.0%) ovaries, 1/4 (25.0%) testes, and in the only epididymis available for assessment (Table 7). In the ovary, immunoreactivity was observed in the interstitium, theca externa, granulosa cells, and rarely within primordial follicles^16^ (Fig. 4h). When available for assessment (3 birds positive in the gonads by RT-qPCR and IHC), no reactivity was observed in the epithelium of the oviduct. Rarely, immunoreactivity was seen in the interstitial cells of the testes (Fig. 4i), and in scattered ciliated epithelial cells in the epididymis of one bird. No reactivity was seen in the epithelium of the seminiferous tubules.

**Table 7:** Immunoreactivity to ABBV-1 N protein in the gonads of Pekin ducks inoculated with aquatic bird bornavirus-1 (ABBV-1) through the intracranial (IC) route and sampled at 21 weeks postinfection.

Duck ID*	Immunoreactivity		Cells positive for ABBV-1 antigen
Females	Ovary	Oviduct	
251	+	−	Interstitial cells in the cortex, primordial follicles
253	+	NA	Granulosa cells
260	+	NA	Interstitial cells in the cortex
266	+	NA	Interstitial cells in the cortex, granulosa cells
268	+	NA	Interstitial cells in the cortex
278	+	−	Interstitial cells in the cortex, granulosa cells
280	+	NA	Interstitial cells in the cortex, theca externa
282	+	−	Interstitial cells in the cortex, granulosa cells

Males	Testes	Epididymis	
259	−	NA	–
263	−	NA	–
269	+	+	Interstitium of the testes, rare ciliated epithelial cells in the epididymis
279	−	NA	–

NA indicates tissue unavailable for analysis.

*No tissues were available for histological assessment for duck 258 (female) and 274 (unknown sex). All other tissues listed in this table were positive for presence of ABBV-1 RNA.

## Discussion

This study documents the successful experimental infection of Pekin ducks with ABBV-1. The ability of the virus to infect another waterfowl species (Muscovy ducks) had been previously documented by our research group^14^. Like in Muscovy ducks, inoculation of the virus via the IC and IM routes in the present study was highly efficient, leading to a 93.3% and 76.7% infection rates, respectively. However, while oral inoculation of both Muscovy ducks and chickens with ABBV-1 did not result in infection^14,15^, choanal inoculation (another mucosal route) led to infection in 4 Pekin ducks, albeit only one of these birds had widespread presence of ABBV-1 in multiple tissues, as shown by RT-qPCR and IHC. This suggests that the upper respiratory tract is a possible, although inefficient, route of infection for ABBV-1, as also shown with PaBVs^18,19^. It appears that parenteral (such as intracranial and intramuscular) routes of inoculation remain the most effective methods to infection for orthobornaviruses, at least in experimental settings^14,15,18,20–22^.

The sequence of positive tissues over time indicates both centrifugal (brain to spinal cord, proventriculus, kidneys, and gonads) and centripetal (muscle to spinal cord and brain) virus spread in both the IC and IM groups, respectively, as also seen for ABBV-1 in chickens and Muscovy ducks, and PaBVs in psittacines^14,15,23,24^. This suggests intra-axonal virus trafficking, as proven with BoDV-1 ^25^, especially in light of the fact that viremia was not detected in our Pekin ducks.

While the average magnitude of virus RNA concentration in tissues increased over time in the IC group, in the IM group, there was a slight decrease in the average virus RNA concentration between 12 and 21 wpi in the brain, spinal cord, and gonads. The reasons for this remain unclear. It could indicate variability of inoculation efficiency in the muscle compared to the intracranial route, or a delayed centripetal spread of infection in certain individual birds. Alternatively, it could point to a slow, progressive clearance of the virus, which was not observed in Muscovy ducks^14^, possibly because of inherent differences between species, or the longer duration (almost double) of the current infection trial.

Immunohistochemistry showed presence of ABBV-1 N protein in multiple tissues of IC and IM birds at 21 wpi, and in the single CH duck also at 21 wpi that was positive by RT-qPCR across multiple tissues. Similar wide tissue distribution of the virus has been documented in natural and experimental ABBV-1 infections in waterfowl^8,14^ and in psittacine birds experimentally infected with PaBV^23,24,26^. In the nervous tissue, immunoreactivity was not associated with areas of inflammation, and appeared multifocally and randomly distributed. No immunoreactivity was observed in the kidneys, despite high virus RNA concentrations. This finding is similar to what observed in Muscovy ducks^14^, and in disagreement with what reported in psittacine birds infected with PaBVs^24,27^. Lack of ABBV-1 immunoreactivity in the kidney is consistent with the low number of positive cloacal swabs (< 0.01% of the total) seen in our cohort. Presence of virus RNA without ABBV-1 N protein expression may be a reflection of the higher sensitivity of RT-qPCR compared to IHC, or indicate tissue-specific downregulation of virus protein expression.

The frequency and magnitude of RNA virus concentration in oral swabs was higher in the IC group when compared to the IM group. The reason for such difference could be related to virus trafficking from the brain through the cranial nerves to the oral mucosa or salivary glands, a path that would require longer for the IM group (inoculated in the leg), compared to the IC ducks. Additionally, as only one cloacal swab was positive (with low virus RNA concentration) in the IC group, oral shedding appears to be a more relevant transmission pathway compared to shedding through the urofeces. One CH duck tested weakly positive in the oral swab at 21 wpi, although this bird was negative in all other tested tissues. The reason for this is unclear, it may be due to contamination of the oral cavity of with low-level ABBV-1 RNA present in the enclosure.

Low concentrations of viral RNA were present in the drinking water of the IC and IM groups only, with higher titers being recorded in the IC group. This is consistent with virus RNA quantification in the oropharyngeal swabs. Although it has been shown that ABBV-1^14^ and PaBVs^22,28^ can be shed intermittently, we detected viral RNA in the drinking water at all tested time points after 10 wpi, likely as a result of virus RNA accumulation in the trough fitted to the automatic waterer. If the virus could survive short periods of time in the environment, its presence in the water (perhaps nearby areas of congregation) could favor infection through the upper respiratory mucosa, or via skin wounds, a newly proposed route of infection for PaBV^29^. We attempted to isolate virus from environmental samples, but this was unsuccessful due to the technical difficulty associated with water filtration to allow isolation in cells (data not shown).

All embryos derived from eggs laid during the experiment tested negative for ABBV-1 RNA. When considering the IC group alone, where 100.0% of females and males were positive for ABBV-1 RNA in the gonads, our results suggest that vertical transmission of ABBV-1 is uncommon. Lack of vertical transmission in face of high infection rate of the gonads could be the consequence of ABBV-1 tissue localization, which appears to be predominately in the interstitial, theca, and granulosa cells, while only rarely in the germinal cells. Vertical transmission could be severely hampered by lack of germ-cell infection, as orthobornaviruses establish persistent infection in the nucleus with little extracellular release^30^. Progression of infection is also an important consideration, since it is impossible to determine when a specific duck developed gonadal infection between 12 and 21 wpi, albeit almost 90% of gonads showed presence of ABBV-1 RNA already at 12 wpi in the IC group. Lastly, one cannot confirm whether infected follicles ovulate or undergo regression. Since the ratio of fertile to total laid eggs, as well as the normalized number of eggs did not differ between groups, it appears that ABBV-1 infection did not markedly affect reproductive physiology. In a previous study in free-ranging Canada geese, out of 22 embryos, 23 hatchlings, and 5 non-embryonated eggs, only one non-embryonated egg had evidence of ABBV-1 RNA in its contents^31^, corroborating the notion that vertical transmission—if possible—is a rare occurrence.

Most common microscopic lesions consisted of lymphocytic perivascular cuffs randomly distributed throughout the central nervous system, similar to what observed with ABBV-1 experimental infections in Muscovy ducks^14^ and natural infections in other waterfowl^8^. These lesions were present with highest frequency in the IC and IM groups, while only 1 infected CH bird presented inflammatory lesions. In both the IC and IM groups, the frequency and severity of encephalitis and/or myelitis peaked at 12 wpi and subsided by 21 wpi. This decline occurred despite birds with inflammatory lesions being still infected (i.e., presence of virus RNA in the brain and/or spinal cord), and even showing a statistically significant increase in the amount of virus RNA in the brain and the spinal cord (from 12 to 21 wpi), as seen in the IC group. These data are in agreement with our previous study in Muscovy ducks^14^ where peak of inflammation was observed at 4 wpi despite virus RNA concentration being highest at 12 wpi in the brain (end of experiment). This could indicate that, while inflammation is eventually cleared, infection of the CNS persists for longer periods of time. Nonetheless, despite this decline in the intensity of inflammation at later time points, regression analysis showed that inflammation was still positively correlated with the burden of virus RNA copies in the brain.

Neuronal chromatolysis was an unexpected finding; it was present in the brainstem of 4 experimentally inoculated ducks in our study. As chromatolysis was observed only in birds positive for virus RNA, we suspect it might have been caused by ABBV-1 infection, although one CH bird with chromatolysis presented with low levels of virus RNA concentration and lack of IHC reactivity. It is possible that infection might have been cleared and neuronal damage persisted. While neuronal damage is occasionally reported in orthobornavirus infection, lesions are typically characterized by single-cell necrosis, not chromatolysis^32^. Possible differentials for this finding include an unexpected background lesion (as rarely reported in toxicological studies of chickens^33^, or in mammals^34^), infection with avian encephalomyelitis virus (AEV) or thiamine deficiency^35^. Birds with chromatolysis were tested by RT-qPCR and resulted negative for AEV (data not shown). Thiamine deficiency is characterized by degenerative lesions scattered throughout the spinal cord and axonal degeneration^35^, which was not observed in our case, as lesions were only confined to rare, scattered neurons in the brainstem. Furthermore, all birds were fed a balanced commercial diet, and it would be difficult to justify why only infected birds would be susceptible. At this point, the significance of chromatolysis remains unclear.

Eight ducks presented neurological signs after 12 wpi. This corresponds to 11.6% and 26.1%, respectively, of all inoculated and IM ducks between 12 and 21 wpi. The observed neurological signs included head bobbing, ataxia, and inability to walk. While similar symptoms have been associated with natural ABBV-1 infection in waterfowl^8^, experimental infection of Muscovy ducks and chickens with ABBV-1 failed to reproduce similar signs, except for one chicken with torticollis^14,15^. Logistic regression analysis showed that development of clinical signs in Pekin ducks was not correlated with inflammation of the CNS, in agreement with development of perivascular cuffs in numerous asymptomatic birds in the present and other studies^14,15^. It is unclear why Pekin ducks developed clinical signs, while Muscovy ducks did not. Possible differences in the adaptive, and possibly innate, immune response could explain this outcome, although both species appear highly permissive to ABBV-1 infection. Lastly, ducks infected intramuscularly had a higher degree of clinical signs compared to the IC and CH groups. While the frequency of clinical sign was too low to confidently assess differences, it is possible that the IM route may have led to more severe damage to the spinal cord and brainstem, as the virus travelled centripetally towards the brain. While inflammation severity in the spinal cord was not different between the IC and IM groups, it should be acknowledged that lesions in the spinal cord can be multifocal, and differences might have been missed due to segmental sampling of this organ. It is also important to recognize that the only CH duck presenting with clinical signs showed incongruous infection parameters, such as minimal amount of viral RNA in the spinal cord and not in the brain, lack of inflammation but presence of chromatolysis, and an ELISA OD value only marginally above the threshold. These conflicting results between indicators of infection and clinical disease pose the questions if the latter may not have been associated with ABBV-1, at least for this one bird. While this is a distinct possibility, there was no evidence of other specific causes for development of clinical signs in our study cohort.

On the other hand, PaBVs infection appears to yield a much higher occurrence of disease: psittacine birds experimentally inoculated with PaBV-2 or PaBV-4 through the IM route alone or combined with the oral and/or subcutaneous routes presented clinical signs in up to 100% of infected birds (although substantial variability is observed)^4,5,19,28,36,37^. These differences may be due to species-specific susceptibility to ABBV-1 infection, an intrinsic higher virulence of PaBVs compared to ABBV-1, or age at infection, as shown by a recent study indicating that older cockatiels are more susceptible to development of clinical signs upon PaBV-4 infection (as compared to younger ones), possibly because younger birds may develop immune tolerance^38^.

## Conclusions

Pekin ducks can become infected with ABBV-1 at high rates when inoculated intracranially or intramuscularly. Although possible, infection through the upper respiratory tract is inefficient. Infected ducks developed inflammatory lesions (i.e., perivascular mononuclear cuffs) exclusively in the nervous system, where highest virus RNA concentration was also observed. Of the virus-inoculated ducks, approximately 9% displayed neurological signs; however, neurological symptoms were not correlated with inflammation or magnitude of virus RNA concentration in the nervous tissue. Shedding occurred mainly through the oral mucosa, and allowed for accumulation of viral RNA in the drinking water. Despite the presence of virus RNA in male and female gonads, vertical transmission did not occur. When compared with the results of a similar study done in Muscovy ducks, Pekin ducks appear to be similarly susceptible to infection, although more likely to shed the virus and to develop clinical signs. Overall, this study described for the first-time infection of Pekin ducks with ABBV-1, and suggests that this species is not only susceptible to ABBV-1 infection, but can also promote its environmental dispersal.

## Materials and methods

### Production of infectious inoculum

The ABBV-1 strain for this experiment was initially isolated from the brain of a naturally infected Canada goose (GenBank number MK966418), and propagated in immortalized duck embryo fibroblasts (American Type Culture Collection CCL-141)^13^. The infectious and control inocula were prepared following the same methods used by our research group in previous studies^14,15^. Briefly, for the infectious inoculum, a stock of confluent 100–150 mm dishes of cells persistently infected with CCL-141 was lysed by osmotic shock followed by one cycle of freeze/thaw in an ultra-cold freezer (− 80 °C), cleared from cell debris by centrifugation, precipitated with polyethylene glycol (PEG)-8000, and resuspended in phosphate-buffered saline (PBS) with 1% fetal bovine serum (FBS) to obtain a ratio of supernatant to final resuspended volume of approximately 100:1^14^. The virus stock was titrated in CCL-141 cells by limiting dilution in a 96-well-plate format. As the virus does not cause cytopathic effect, positive wells were detected by immunofluorescence (IFA) using a monospecific antibody directed against the ABBV-1 N protein (Pacific Immunology). The titer was calculated using the Karber’s method, reported as 50% tissue culture infectious dose (TCID_50_)/mL, and converted to focus forming units (FFUs). The control inoculum (carrier) consisted of PBS precipitated with 30% PEG, centrifuged, and then resuspended in PBS with 1% FBS to obtain a ratio of initial PBS to final resuspended volume of approximately 100:1.

### Animal experiment and sample collection

#### Experimental design

A layout of the experimental plan and sampling is provided in Supplementary Fig. 1. The experiment was designed to include 112 birds plus 9 birds to account for unexpected mortality (~ 8% attrition rate). In total, 121 1-day-old Pekin ducks (*Anas platyrhynchos domesticus*) were purchased from a local hatchery (Feathered Acres, Fergus, ON, Canada) and delivered to the Research Isolation Unit of the Central Animal Facility of the University of Guelph. Upon arrival, ducklings were neck tagged and divided into four groups: control (CO; n, 29; 13 males, 15 females, 1 undetermined), intracranial (IC; n, 32; 20 males, 9 females, and 3 undetermined), intramuscular (IM; n, 30; 14 males and 16 females), and choanal (CH; n, 30; 16 males, 13 females, and 1 undetermined). Experimental groups were kept in separate, approximately 6 m^2^ pens with negative pressure, and 12 h of light and 12 h of dark. Ducklings were placed on the floor with shavings, and food and water were offered ad libitum. The water bowls were flushed daily and re-filled automatically. Infrared lamps were provided for optimal temperature until 3 weeks of age.

After 24 h from arrival, ducklings were inoculated with ABBV-1 or control carrier, as described previously^14^. For the intracranial (IC) group, each duck was inoculated with 50 μL of inoculum containing 10^4.16^ FFUs of ABBV-1 directly into the subdural space of the right cerebral hemisphere. Each duck in the intramuscular (IM) and choanal (CH) groups was inoculated with 100 μL of virus inoculum containing 10^4.46^ FFUs of ABBV-1 in the right gastrocnemius muscle or directly into the choanal slit (delivered through the mouth), respectively. All ducks in the control (CO) group were inoculated with carrier only using the same 3 inoculation routes and volumes employed for the infectious inocula.

Following inoculation, ducks were monitored daily for the presence of clinical signs. Ducks with neurological signs, or unable to reach food and water were euthanized regardless of sampling schedule, and were grouped with the subsequent sampling point.

#### Euthanasia and tissue sample collection

From each group, seven ducks at 1 and 12 weeks postinfection (wpi), and 15–18 ducks at 21 wpi were scheduled to be randomly selected and euthanized for sample collection (Supplementary Fig. 1). One-week-old ducklings were anesthetized with isoflurane in a 7 L vented induction chamber (VetEquip©) and euthanized by carbon dioxide (CO_2_) inhalation. Older ducks were euthanized by pentobarbital overdose delivered intravenously (100 mg/kg), after being sedated with intramuscular medetomidine (0.02 mg/kg) and ketamine (30 mg/kg).

Oropharyngeal and cloacal swabs for RNA extraction, and 2 mL of blood (jugular vein) for both RNA extraction and serology were collected from every duck immediately after euthanasia. For each bird, a full postmortem was carried out to assess any macroscopic abnormalities, and select tissues (brain, lumbar spinal cord, proventriculus, ventriculus, kidneys and gonads) were sampled for both RNA extraction and histology (*partial sampling*). For 3–6 ducks from each group at each time point, additional tissues were sampled to increase the granularity of the histology assessment (*full sampling*; see below, “histopathological assessment”). For tissues collected for both RNA extraction and histology, two samples were collected separately; for the brain, after removal of the entire brain from the skull, a portion of approximately one third of the frontal cerebrum (~ 50 mg) was sampled for RNA extraction, while the rest was fixed in formalin. Ducks that died unexpectedly or were euthanized outside of sampling schedule underwent full sampling, and test results were tallied with the next sampling time point for analytical purposes.

#### Assessment of vertical transmission and environmental sampling

Ducks began laying eggs at 16 wpi. Eggs were opportunistically collected daily and kept at room temperature for up to 24 h at the animal facility, until transported to the laboratory. Upon inspection, eggs that were not cracked or heavily contaminated with fecal material were date stamped, incubated in a 1502 Digital Sportsman Incubator (GQF Manufacturing Company Inc.) at 37.7 °C with 80% humidity, and candled daily. Unfertile eggs were discarded, while embryonated eggs were incubated up to 18 to 21 days, and then transferred to 4 °C overnight. Embryos were then extracted and dissected to collect separately the brain and a pool of internal organs (proventriculus, ventriculus, liver, kidney, and heart), which underwent RNA extraction.

Presence of virus RNA in the environment was tested in the drinking water starting at 4 wpi, and then every 2 weeks until the end of the experiment. From each pen, a 1.5 mL volume of drinking water was collected from an automatic waterer fitted with a trough. A sterile disposable pipette was used to collect the water, which was transferred to a sterile 1.5 mL screw cap tube and transported to the laboratory for RNA extraction.

#### Animal use protocol approval

All experimental procedures were approved by the Animal Care and Use Committee of the University of Guelph (Animal Utilization Protocol 3978) and conducted in accordance with the relevant regulations set out by the Canadian Council on Animal Care.

### Histopathological assessment

#### Samples and processing

All tissues sampled during postmortem examination were fixed in 10% neutral buffered formalin and routinely processed for histology (hematoxylin and eosin, H&E). For partial sampling, brain, lumbar spinal cord, proventriculus, ventriculus, kidneys, and gonads were collected. For full sampling, tissues included brain, spinal cord (cervical, thoracic, and lumbar), ischiatic nerves, brachial plexus, right gastrocnemius muscle (only for ducks in the CO and IM groups), trachea, lungs, heart, esophagus, proventriculus, ventriculus, small intestine (at the level of the Meckel’s diverticulum), colon, liver, pancreas, spleen, thymus, cloacal bursa, thyroid glands, adrenal glands, kidneys, gonads, and nasal passages (only for ducks in the CO and CH groups). The nasal passages (proximal bill, including turbinates) were decalcified 24–48 h post fixation using Cal-Ex II Fixative Decalcifier (Fisher Scientific), and sectioned coronally. Coronal sections of the cerebrum, optic lobe, brainstem, and cerebellum, and at least two coronal and/or transverse sections of each segment of the spinal cord were included to examine the central nervous system (CNS).

#### Scoring of microscopic lesions in the central nervous system

Histology slides stained with H&E were examined and scored in a nonblinded fashion by one member of the investigative team (F.A.). Presence of microscopic lesions was tallied nominally for each evaluated tissue. Since mononuclear encephalitis and myelitis were exceedingly the most common lesion, this type of inflammation was evaluated using a semi-quantitative scoring system developed for a similar study in chickens and Muscovy ducks^14,15^, with minor adaptations to better reflect the range of lesions observed in Pekin ducks.

Three different regions of the brain (cerebrum, cerebellum, and brainstem) and the spinal cord (all available sections together, regardless of anatomical segment) were scored separately (i.e., subscore), by assessing the average thickness of the perivascular cuffs (PVC; intensity of inflammation) and the average number of vessels affected (distribution of inflammation) in 10, randomly selected 100 × fields (1.52 mm^2^). Each region received a subscore resulting by adding the distribution score (no PVCs, score 0; 1–5 PVCs, score 1; 6–10 PVCs, score 2; > 11 PVCs, score 3) and intensity score (no inflammation, score 0; < 1.5 layers wide, score 1; 1.5 < 2.0 layers wide, score 2; > 2 layers wide, score 3). The final score for the brain was calculated as the simple average between all assessed brain regions (i.e., average of subscores for cerebrum, brainstem, and cerebellum). For the spinal cord, the final score corresponded to the calculated subscore. For descriptive purposes, lesions were ranked into mild (scores 1–2), moderate (3–4), and severe (5–6).

#### Immunohistochemistry

Virus distribution in tissues and cells was evaluated by immunohistochemistry (IHC), using a rabbit monospecific antibody against the ABBV-1 N protein at 1:6000 dilution, and coupled with a Nova Red chromogen (Vector Laboratories), as previously described^14,15,39^. The IHC test was conducted on all tissues from 3 IC, 3 IM, and 3 CH ducks that underwent full sampling at 21 wpi, as well as the only CH duck at the same time point that showed widespread virus distribution by RT-qPCR and underwent partial sampling. One CO duck from the same collection time point was used as an age-matched, negative control. Additionally, IHC was carried out on the gonads from all IC birds and one CO duck euthanized at 21 wpi, as part of the assessment for vertical transmission (see below). Lastly, for ducks that developed neurological signs outside specific time points, IHC was carried out on both the brain and the spinal cord. The positive control used for the IHC technique consisted of brain tissue from a Canada goose naturally infected with ABBV-1, as identified in a previous study^8^. Only cells that displayed nuclear, or nuclear and cytoplasmic immunoreactivity were considered positive.

### Quantification of virus RNA in tissues, swabs, drinking water, and embryos

Tissues samples from birds (brain, spinal cord, proventriculus, kidney, and gonads), embryos (brain and pooled tissues), and swabs were placed into separate sterile screw cap tubes containing 0.7 mL of preserving solution (20 mM ethylenediaminetetraacetic acid [EDTA], 25 mM sodium citrate, and 70% (w/v) ammonium sulfate with a pH of 5.2) and stored at − 80 °C until processing. Drinking water samples were directly placed into the ultracold freezer, without preservatives. Total RNA was extracted from approximately 300 μg of tissue and 150 μL of swab liquid or drinking water, using the E.Z.N.A® RNA Kit II or the Viral RNA Kit, respectively (both from Omega Bio-Tek). The purified RNA was used to amplify the target region of the ABBV-1 N gene using primers and fluorescent probes coupled with the Luna® Universal Probe One-Step reverse transcription quantitative polymerase chain reaction (RT-qPCR) kit (New England Biolabs), as described previously^13^. For tissues, copy numbers of virus RNA (per 150 ng of total tissue RNA) were calculated by interpolating the threshold (Ct) value of the samples against a standard curve made of serial dilutions of a 500 bp gBlock gene fragment of the ABBV-1 N gene, which was run with each plate^14^. For swabs and water, the standard curve was made by extracting RNA of a serially diluted stock of known amount of virus in water, and the titre was expressed as focus forming units (FFUs) RNA equivalent per μL. All samples were run in triplicates wells using the Roche LightCycler® System; samples were considered positive if the cycle threshold (Ct) was < 35.

### Serology

Following the euthanasia of each duck, blood was immediately collected from the jugular vein and allowed to coagulate for up to 8 h. Sera were separated by centrifugation (2000×*g* for 10 min) and used to assess presence of antibodies by enzyme-linked immunosorbent assay (ELISA) across all groups and time points, as previously described^14,15^. Briefly, wells of 96-well microtiter ELISA plates (ThermoFisher) were coated using a recombinant full-length ABBV-1 N protein (Biomatik) at 25 ng/well at 4 °C, subsequently blocked with 5% fish gelatin in PBS overnight at 4 °C, and then incubated for 2 h at 37 °C with 50 μL of duck sera diluted 1:200 in PBS-T with 1% fish gelatin. After abundant washes (3 times with PBS-T and 2 times with water), a secondary HRP-conjugated goat anti-avian IgY (heavy and light chains, Cedarlane) was applied at 1:10,000 dilution for 2 h at 37 °C. Following additional abundant washes, binding was detected by adding 50 μL/well of chromogenic substrate (Pierce 1-Step Ultra TMB-ELISA Substrate Solution; ThermoFisher). The optical density (OD) of the reaction was read at a wavelength of 450 nm, using an EnSpire multimode plate reader (Perkin Elmer).

To decrease the variability of readings between plates, one sample that had moderate to high reactivity was run in parallel for every plate (i.e., calibrator). Calibration was carried out by dividing the raw OD values of each sample on a plate by the OD of its calibrator and multiplying by the average of all calibrators across plates. The cut-off for positive sera was determined as the average of all the CO sera + 3 times the standard deviation (SD). All samples and calibrators were run in triplicate, and calculations were conducted on the averages of the 3 wells.

### Statistical analysis

#### Descriptive statistics

Differences in the frequency of inflammation between different areas of the CNS within each experimental group (IC, IM, and CH) was evaluated by pairwise comparison for proportions and Holm’s tests correction for multiple comparisons. Differences in the average severity of CNS inflammation scores, as well differences between average OD ELISA values were tested by Kruskal–Wallis test with Dunn’s test for multiple comparisons. A two-way analysis of variance (ANOVA) with Tukey’s post-hoc test was used to evaluate differences between the average concentration of virus RNA copy number in the tissues of infected ducks, with inoculation route and time point as explanatory variables (main effects model). The daily number of eggs laid across experimental groups was compared with a Kruskal–Wallis test, after normalizing by the number of females (as assessed during necropsy) in each group.

#### Multivariable regression analysis

A multivariable logistic regression model was used to predict the occurrence of neurological signs (categorical; dependent variable), using as explanatory variables time postinfection (binary variable; 12 and 21 wpi), severity of inflammation (continuous; pathology subscores for cerebrum, cerebellum, and spinal cord), virus RNA copies in the brain and spinal cord (continuous), serology (OD) values (continuous), and inoculation route (categorical; IC and IM). A second multivariable logistic regression model was used to predict inflammation of the CNS (categorical variable; presence/absence) using time postinfection (binary; 12 and 21 wpi), virus RNA copies in brain and spinal cord (continuous), serology (OD) values (continuous), and inoculation route (categorical) as explanatory variables. Presence of chromatolysis was not included due to the low frequency of this variable in the cohort.

Independent variables were included into the multivariable models if they were shown to be significantly associated with the dependent variable using univariable logistic models with a relaxed p-value (p ≤ 0.2). Collinearity between explanatory variables was tested by pairwise Spearman’s correlation tests: when two variables were highly correlated (rho > 0.70; p < 0.05), only the one with the smallest p-value was considered for inclusion in the multivariable models. The virus RNA concentration was expressed as a log10 copy number per 150 ng of total RNA, and any zero values were changed to 1.0 × 10^–5^. Only day from 12 and 21 wpi were included, and for each univariable logistic model, 82 observations were present.

Differences in the proportion between fertile over total eggs laid in each group were tested by a chi-square test. Statistical analysis was carried out using R software (R Core Team, 2023) for comparison of proportions and regression analyses; GraphPad Prism for iOS, version 9 (GraphPad Software, La Jolla, USA), was used for all the other tests. Significance for each test was set at *p* < 0.05.

### Ethical approval

Animal use and experimental procedures involving animals were approved by the University of Guelph Animal Care and Use Committee (Animal Utilization Protocol #3978). The study is reported in accordance with ARRIVE guidelines (https://arriveguidelines.org).

### Supplementary Information

Supplementary Information.

## Data Availability

All data generated or analysed during this study are included in this published article and its Supplementary Information files.

## References

[1] Walker PJ (2022). Recent changes to virus taxonomy ratified by the International Committee on Taxonomy of Viruses (2022). Arch. Virol..

[2] Honkavuori KS (2008). Novel borna virus in psittacine birds with proventricular dilatation disease. Emerg. Infect. Dis..

[3] Kistler AL (2008). Recovery of divergent avian bornaviruses from cases of proventricular dilatation disease: Identification of a candidate etiologic agent. Virol. J..

[4] Gancz AY (2009). Experimental induction of proventricular dilatation disease in cockatiels (*Nymphicus hollandicus*) inoculated with brain homogenates containing avian bornavirus 4. Virol. J..

[5] Gray P (2010). Use of avian bornavirus isolates to induce proventricular dilatation disease in conures. Emerg. Infect. Dis..

[6] Delnatte P (2011). New genotype of avian bornavirus in wild geese and trumpeter swans in Canada. Vet. Rec..

[7] Payne S (2011). Detection and characterization of a distinct bornavirus lineage from healthy Canada geese (*Branta canadensis*). J. Virol..

[8] Delnatte P (2013). Pathology and diagnosis of avian bornavirus infection in wild Canada geese (*Branta canadensis*), trumpeter swans (*Cygnus buccinator*) and mute swans (*Cygnus olor*) in Canada: A retrospective study. Avian Pathol..

[9] Delnatte P (2014). Avian bornavirus in free-ranging waterfowl: Prevalence of antibodies and cloacal shedding of viral RNA. JWDI.

[10] Guo J (2012). Widespread avian bornavirus infection in mute swans in the Northeast United States. VMRR.

[11] Payne SL (2012). Birds and bornaviruses. Anim. Health Res. Rev..

[12] Nielsen AMW, Ojkic D, Dutton CJ, Smith DA (2018). Aquatic bird bornavirus 1 infection in a captive Emu (*Dromaius novaehollandiae*): Presumed natural transmission from free-ranging wild waterfowl. Avian Pathol..

[13] Pham PH, Leacy A, Deng L, Nagy É, Susta L (2020). Isolation of Ontario aquatic bird bornavirus 1 and characterization of its replication in immortalized avian cell lines. Virol. J..

[14] Iverson M (2022). Experimental infection of aquatic bird bornavirus in Muscovy ducks. Sci. Rep..

[15] Iverson M (2022). Experimental infection of aquatic bird bornavirus 1 in domestic chickens. Vet. Microbiol..

[16] Siegel PBH, Christa F, Scanes CG, Scanes CG, Dridi S (2022). Domestication of poultry. Sturkie’s Avian Physiology.

[17] Chen X (2021). Centennial Review: History and husbandry recommendations for raising Pekin ducks in research or commercial production. Poult. Sci..

[18] Heckmann J (2017). Investigation of different infection routes of parrot bornavirus in cockatiels. Avian Dis..

[19] Rubbenstroth D (2022). Avian bornavirus research—A comprehensive review. Viruses.

[20] Piepenbring AK (2012). Pathogenesis of avian bornavirus in experimentally infected cockatiels. Emerg. Infect. Dis..

[21] Piepenbring AK (2016). Parrot Bornavirus (PaBV)-2 isolate causes different disease patterns in cockatiels than PaBV-4. Avian Pathol..

[22] Runge S (2017). Viral vector vaccines protect cockatiels from inflammatory lesions after heterologous parrot bornavirus 2 challenge infection. Vaccine.

[23] de Araujo JL (2017). From nerves to brain to gastrointestinal tract: A time-based study of parrot bornavirus 2 (PaBV-2) pathogenesis in cockatiels (*Nymphicus hollandicus*). PLoS ONE.

[24] Petzold J (2022). Tissue distribution of parrot bornavirus 4 (PaBV-4) in experimentally infected young and adult cockatiels (*Nymphicus hollandicus*). Viruses.

[25] Carbone KM, Duchala CS, Griffin JW, Kincaid AL, Narayan O (1987). Pathogenesis of Borna disease in rats: Evidence that intra-axonal spread is the major route for virus dissemination and the determinant for disease incubation. J. Virol..

[26] de Araujo JL (2019). Distribution of viral antigen and inflammatory lesions in the central nervous system of cockatiels (*Nymphicus hollandicus*) experimentally infected with parrot bornavirus 2. Vet. Pathol..

[27] Raghav R (2010). Avian bornavirus is present in many tissues of psittacine birds with histopathologic evidence of proventricular dilatation disease. J. Vet. Diagn. Investig..

[28] Payne S (2011). Unusual and severe lesions of proventricular dilatation disease in cockatiels (*Nymphicus hollandicus*) acting as healthy carriers of avian bornavirus (ABV) and subsequently infected with a virulent strain of ABV. Avian Pathol..

[29] Heckmann J (2020). Wounds as the portal of entrance for parrot bornavirus 4 (PaBV-4) and retrograde axonal transport in experimentally infected cockatiels (*Nymphicus hollandicus*). Avian Dis..

[30] Matsumoto Y (2012). Bornavirus closely associates and segregates with host chromosomes to ensure persistent intranuclear infection. Cell Host Microbe.

[31] Delnatte P, Nagy E, Ojkic D, Crawshaw G, Smith DA (2014). Investigation into the possibility of vertical transmission of avian bornavirus in free-ranging Canada geese (*Branta canadensis*). Avian Pathol..

[32] Tizard I, Ball J, Stoica G, Payne S (2016). The pathogenesis of bornaviral diseases in mammals. Anim. Health Res. Rev..

[33] Bickford AA, Sprague GL (1982). Critical neurohistopathologic evaluation of clinically healthy commercial single-comb White Leghorn hens. Avian Dis..

[34] Wohlsein P, Deschl U, Baumgärtner W (2013). Nonlesions, unusual cell types, and postmortem artifacts in the central nervous system of domestic animals. Vet. Pathol..

[35] Crespo R, Shivaprasad HL, Saif YM (2013). Developmental, metabolic, and other noninfectious disorders. Disease of Poultry.

[36] Mirhosseini N (2011). Proventricular dilatation disease in cockatiels (*Nymphicus hollandicus*) after infection with a genotype 2 avian bornavirus. J. Avian Med. Surg..

[37] Rubbenstroth D (2014). No contact transmission of avian bornavirus in experimentally infected cockatiels (*Nymphicus hollandicus*) and domestic canaries (*Serinus canaria* forma domestica). Vet. Microbiol..

[38] Gartner AM (2021). Age-dependent development and clinical characteristics of an experimental parrot bornavirus-4 (PaBV-4) infection in cockatiels (*Nymphicus hollandicus*). Avian Pathol..

[39] Leacy A, Nagy É, Pham PH, Susta L (2020). In vitro and in ovo host restriction of aquatic bird bornavirus 1 in different avian hosts. Viruses.

