# Supplementary material for: Experimental pathogenesis of aquatic bird bornavirus 1 in Pekin ducks
Source: Sci Rep. 2023 Oct 23;13:18094. doi: 10.1038/s41598-023-45205-0 (PMC10593797; doi:10.1038/s41598-023-45205-0)
Supplement: Supplementary file 1 — Supplementary Information. [file 41598_2023_45205_MOESM1_ESM.pdf]

SUPPLEMENTARY MATERIAL:

Fernanda Ampuero[a], Alexander Leacy[a], Phuc H. Pham[a], Sunoh Che[a], Claire Jardine[a], Eva Nagy[a], Pauline Delnatte[a], Brandon Lillie[a], Leonardo Susta[a]*

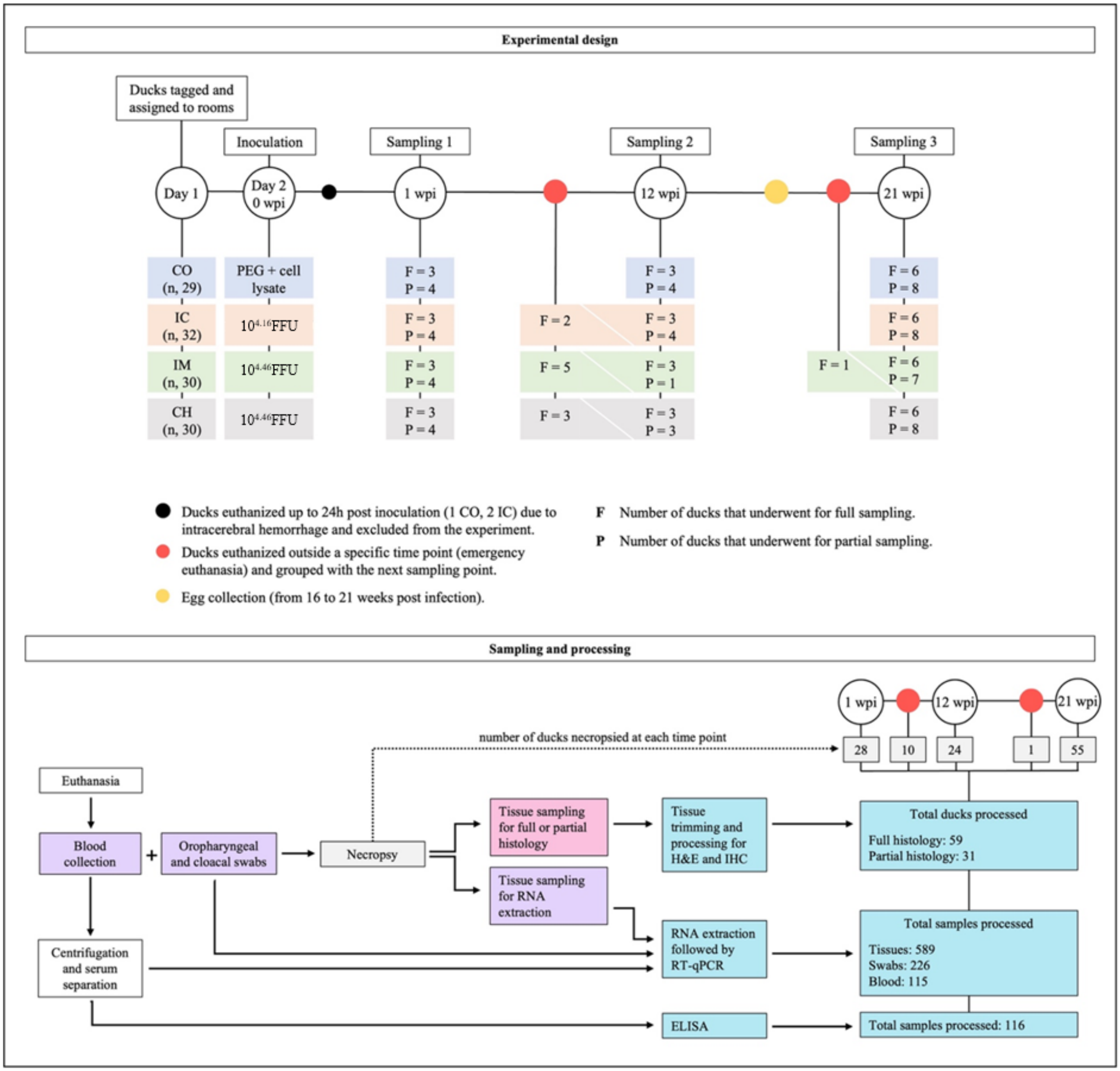

Supplementary Table 1: Number of eggs laid per day by Pekin ducks inoculated with aquatic bird bornavirus 1 (ABBV-1) through the intracranial (IC), intramuscular (IM), and choanal (CH) routes and the control (CO) group, as assessed between the first and last day of lay for each group.

**Day**	1[§]	2	3	4	5	6	7	8	9	10	11	12	13	14	15	16	17	18	19	20	21	22	23	24	25	26	27	28	29	30	31	32	33	34[§]
**CO (8)***	-	-	-	-	-	-	-	-	-	1	1	1	1	1	2	2	2	2	4	3	5	6	7	5	6	6	5	9	6	7	6	0	0	0
**IC (9)**	-	-	-	1	0	1	1	2	1	1	0	0	0	0	1	1	1	1	0	0	2	2	2	1	3	2	5	4	5	4	6	6	7	0
**IM (5^)**	1	1	1	0	0	1	0	1	1	1	1	0	1	1	1	1	0	1	1	1	1	1	1	0	0	2	1	1	2	1	3	2	2	2
**CH (6)**	-	-	1	0	0	0	0	1	0	0	0	0	1	2	2	3	3	3	2	3	8	1	3	2	3	3	4	3	5	3	3	2	0	0

*Indicates the number of hens in each group, as assessed at necropsy.

[§]The first day in the table corresponds to day 115 postinfection; the last day is the day of necropsy (day 148 postinfection).

^ One less hen after day 11 (euthanized).

- Indicates days before the beginning of lay for that group.

Supplementary Table 2. Number of tissues positive for ABBV-1 RNA in each group of Pekin ducks inoculated with aquatic bird bornavirus 1 (ABBV-1) through the intracranial (IC), intramuscular (IM), and choanal (CH) routes, and the control (CO) group at 21 weeks postinfection.

**Tissue**	**CO**	**IC***	**IM**	**CH**
Ovary	0/8	8/9	1/5	1/6
Testes	0/6	3/4	4/9	0/8

*For one additional IC bird (#274), sex could not be determined and the tissue was not included in the tally.
